# Establishment of an immortalized porcine erythroid progenitor cell line using species-adapted papillomavirus oncogenes

**DOI:** 10.3389/fbioe.2026.1807068

**Published:** 2026-08-26

**Authors:** Tra My Tran, Hyeon Ho Lee, Tae Hyeon Hwang, Min Hyuk Kim, Nhat Minh Dang, Min Guk Lee, Joohyun Shim, Jeong Ho Hwang, Jae Young Kim

**Affiliations:** 1 Department of Life Science, Gachon University, Seongnam, Republic of Korea; 2 Department of Transgenic Animal Research, Optipharm, Inc., Cheongju-si, Republic of Korea; 3 Division of Advanced Predictive Research, Center for Bio-Signal Research, Korea Institute of Toxicology (KIT), Daejeon, Republic of Korea

**Keywords:** cell line, erythropoiesis, hematopoietic stem cells, lentivirus, swine

## Abstract

**Introduction:**

Critically low blood donation rates and recurrent shortages in the blood supply have become pressing challenges in transfusion medicine. Xenotransfusion using porcine red blood cells (pRBCs) represents a promising alternative; however, risks of immune rejection and infection necessitate the development of genetically engineered porcine models – a process that is time-consuming, costly, and technically complex. In this study, we established an immortalized porcine erythroid progenitor cell line as a platform for genetic manipulation and the evaluation of xenotransfusion potential.

**Methods:**

Porcine hematopoietic progenitor cells (CD117^+^) were isolated from bone marrow mononuclear cells and immortalized via lentiviral transduction with E6/E7 oncogenes from Human papillomavirus type 16 (HPV16) and Sus scrofa papillomavirus (SPV2).

**Results:**

The resulting cells were optimized for long-term culture and exhibited stable proliferation. The immortalized porcine erythroid progenitor cells retained the capacity to differentiate into red blood cells.

**Discussion:**

Our findings demonstrate that E6/E7-mediated immortalization facilitates the generation of porcine erythroid progenitor cells capable of differentiating into red blood cells, offering a valuable tool for genetic engineering and a promising avenue toward safe, sustainable heterologous blood transfusion strategies.

## 1 Introduction

The persistent decline in blood donation rates, combined with frequent shortages of blood products, poses a critical challenge to modern transfusion medicine. Heightened concerns about the transmission of infectious diseases have tightened donor eligibility criteria, further exacerbating the limited availability of human red blood cells (hRBCs) (Ellingson et al., 2017). While alternatives such as hemoglobin-based oxygen carriers and stem cell-derived RBCs have been explored, they have achieved limited clinical success (Trakarnsanga et al., 2017; Douay, 2018).

Porcine red blood cells (pRBCs) have emerged as a potential xenotransfusion candidate due to pigs’ physiological similarity to humans, high reproductive rate, ease of rearing, and amenability to genetic modification (Zhu, 2000; Butler et al., 2015). Furthermore, porcine hemoglobin shares a high degree of structural and sequence similarity with human hemoglobin, and pigs can be raised under pathogen-free conditions, mitigating infection risks (Smood et al., 2019). Recent progress in xenotransplantation has highlighted the feasibility of using genetically modified pigs to overcome immunological barriers, such as hyperacute rejection mediated by antigens such as α-Gal, Neu5Gc, and Sda (Wang et al., 2014; Park et al., 2023).

Several studies have demonstrated the successful establishment of immortalized erythroid progenitor cell lines from various sources, including cord blood, peripheral blood mononuclear cells (PBMCs), and induced pluripotent stem cells (iPSCs) (Trakarnsanga et al., 2017; Kurita et al., 2013). However, these cell lines often exhibit limitations such as low hemoglobin synthesis and poor enucleation efficiency, which hinder their translational application for transfusion purposes. In contrast, erythroid cell lines derived from bone marrow CD34^+^ hematopoietic progenitor cells have demonstrated superior hemoglobin production and enucleation capacity (Kurita et al., 2013; Leberbauer et al., 2005). In the context of porcine models for xenotransfusion, we observed that CD117^+^ cells sorted from porcine bone marrow also co-express CD34, suggesting their potential as erythroid progenitors. While anti-pig CD117 antibodies are commercially available and have been effectively used for porcine cell isolation (Pérez et al., 2007), reliable anti-pig CD34 antibodies have not yet been widely developed, presenting a limitation in isolating pure CD34^+^ populations in swine. Nevertheless, CD117^+^ cells from pig bone marrow have already been applied in studies of xenotransfusion, making them a practical and accessible target for immortalization (Ohshima et al., 2014).

Establishing immortalized porcine erythroid progenitor cell lines presents a valuable alternative to transgenic pig models by enabling high-throughput *in vitro* analyses of immunological barriers, such as phagocytosis and hyperacute rejection. While immortalization using E6/E7 oncoproteins from human papillomavirus (HPV) has been well documented in human systems, porcine cells have shown species-specific resistance to these methods, requiring tailored approaches (Kurita et al., 2013; Scully et al., 2019). Recent identification of *Sus scrofa* papillomavirus 2 (SPV2), a swine-specific papillomavirus, provides a novel tool for porcine cell immortalization (Link et al., 2017). In this study, we report the successful generation of the first immortalized adult porcine erythroid cell line using CD117^+^ hematopoietic progenitor cells transduced with both SPV2 E6/E7 and HPV16 E6/E7 via lentiviral vectors. The resulting cells demonstrated stable long-term proliferation and offer a promising *in vitro* platform for genetic manipulation, functional characterization, and immune compatibility testing in xenotransfusion research.

## 2 Materials and methods

### 2.1 Lentiviral vector construction for SPV2 E6/E7 expression

The coding sequence for *Sus scrofa* papillomavirus 2 (SPV2) E6/E7 was amplified using Phusion™ Plus PCR Master Mix (Thermo Fisher Scientific). The HPV16 E6/E7 fragment in the CSIV-TRE-HPV16-E6/E7-UbC-KT lentiviral vector (#17719B0G1, RIKEN BRC) was replaced with the SPV2 E6/E7 insert using T4 DNA ligase (New England Biolabs), generating the CSIV-TRE-SPV2-E6/E7-UbC-KT construct. This vector was then used for lentiviral production and transduction of porcine hematopoietic progenitor cells to establish the immortalized erythroid cell line.

### 2.2 Establishment of immortalized porcine erythroid line from CD117+ HSPCs

Bone marrow mononuclear cells were obtained from a GGTA1/CMAH/B4galNT2/A3GalT2 Knockout and human CD46/TBM Knockin miniature male pig provided by Optipharm Inc. (Cheongju, Korea), and CD117^+^ hematopoietic progenitor cells were enriched using magnetic-activated cell sorting (MACS). After Fc receptor blocking (Miltenyi Biotec), cells were incubated with an anti-pig CD117 monoclonal antibody (MyBioSource) and then labeled with Anti-Mouse IgG MicroBeads (Miltenyi Biotec). Labeled cells were passed through MACS columns to positively isolate CD117^+^ cells. The cells were cultured in a stimulation medium (Online Supplementary Table S1). After 24 h of pre-stimulation, lentiviral transduction was performed using vectors encoding SPV2 E6/E7 or HPV16 E6/E7, in the presence of 8 μg/mL polybrene (Santa Cruz Biotechnology). The cells were transferred to an expansion medium (Supplementary Table S1), and supplemented with 1 μg/mL doxycycline to induce E6/E7 gene expression under the Tet-inducible system.

To induce RBC maturation, the erythroid line was first cultured in stimulation medium in the presence of 1 μg/mL doxycycline and maintained for 6 days at a density of 2–3 × 10^5^ cells/mL. On day 6, the culture was switched to differentiation medium (DM; Online Supplementary Table S1). Cells were maintained in DM until day 18 to support full erythroid differentiation and hemoglobinization.

### 2.3 Preparation of RNA and reverse transcription-quantitative (RT-qPCR)

Total RNA was extracted from cultured cells, primary CD71^+^ erythroid progenitor cells isolated from bone marrow mononuclear cells of GGTA1/CMAH/B4GalNT2/A3GalT2 Knockout and human CD46/TBM Knockin miniature pigs. The first-strand cDNA was generated using the ReverTra Ace™ qPCR RT Kit (TOYOBO). Quantitative PCR was then performed with SYBR® Green Realtime PCR Master Mix (TOYOBO) on a LineGene 9600 Plus real-time system (BIOER, Hangzhou, China) with the following primer pairs (Supplementary Table S2).

### 2.4 Flow cytometry

Flow cytometry was performed to assess transduction efficiency (hKO1 fluorescence) and surface marker expression. Cells were collected, washed with DPBS +1% BSA, and stained with primary antibodies against CD117, CD36, CD71, CD34, RhD, and Neu5Gc or primary Isolectin GS-IB4 specific for α-Gal epitope followed by fluorescent-conjugated secondary antibodies, Hoechst staining was performed to assess the enucleation efficiency of differentiated cells, the information of the antibodies and lectin are described in detail in the Supplementary Table S3. Samples were analyzed using a Cytomics FC500 MLP (Beckman Coulter) or BD Accuri™ C6 Plus (BD Biosciences).

For the analysis of human natural antibody binding, cells were incubated with 25% human serum in PBS for 30 min. Cells were washed and stained with PerCP-Cy5.5-conjugated antibodies specific for human IgG Fc or human IgM Fc for 1 h. The binding levels of human IgG and IgM on the cell surface were determined by flow cytometric analysis.

### 2.5 Cell morphology analysis

Cell morphology and maturation were assessed by Giemsa staining for 5 min followed by 5 min Wright-Giemsa staining. After rinsing with distilled water and air-drying, slides were examined using a bright-field microscope (Olympus CKX53, Tokyo, Japan).

Hemoglobin synthesis and enucleation during erythropoiesis were assessed by benzidine-peroxidase and Hoechst staining. Cells were fixed in 10% formaldehyde-methanol for 1 min and stained with a benzidine-peroxidase solution containing 3,3′-diaminobenzidine and hydrogen peroxide (H_2_O_2_) diluted in PBS for 10 min. Cells were then stained with Hoechst for 20 min to distinguish nucleated and enucleated cells. The stained slides were then observed using a fluorescence microscope (Olympus CKX53, Tokyo, Japan).

### 2.6 Hemoglobin-oxygen dissociation analysis

Deoxygenation of hemoglobin after differentiation of the erythroid progenitor cell lines was assessed by incubation with sodium metabisulfite (Na_2_S_2_O_5_) at various concentrations, thereby inducing conformational changes in hemoglobin tertiary structure. Resulting spectral shifts were quantified by measuring absorbance from 380 to 450 nm using a microplate reader (µ-Quant, BioTek Instruments, Winooski, USA).

## 3 Results

### 3.1 Enrichment and characterization of CD117+ cells from porcine bone marrow mononuclear cells

**FIGURE 1:**
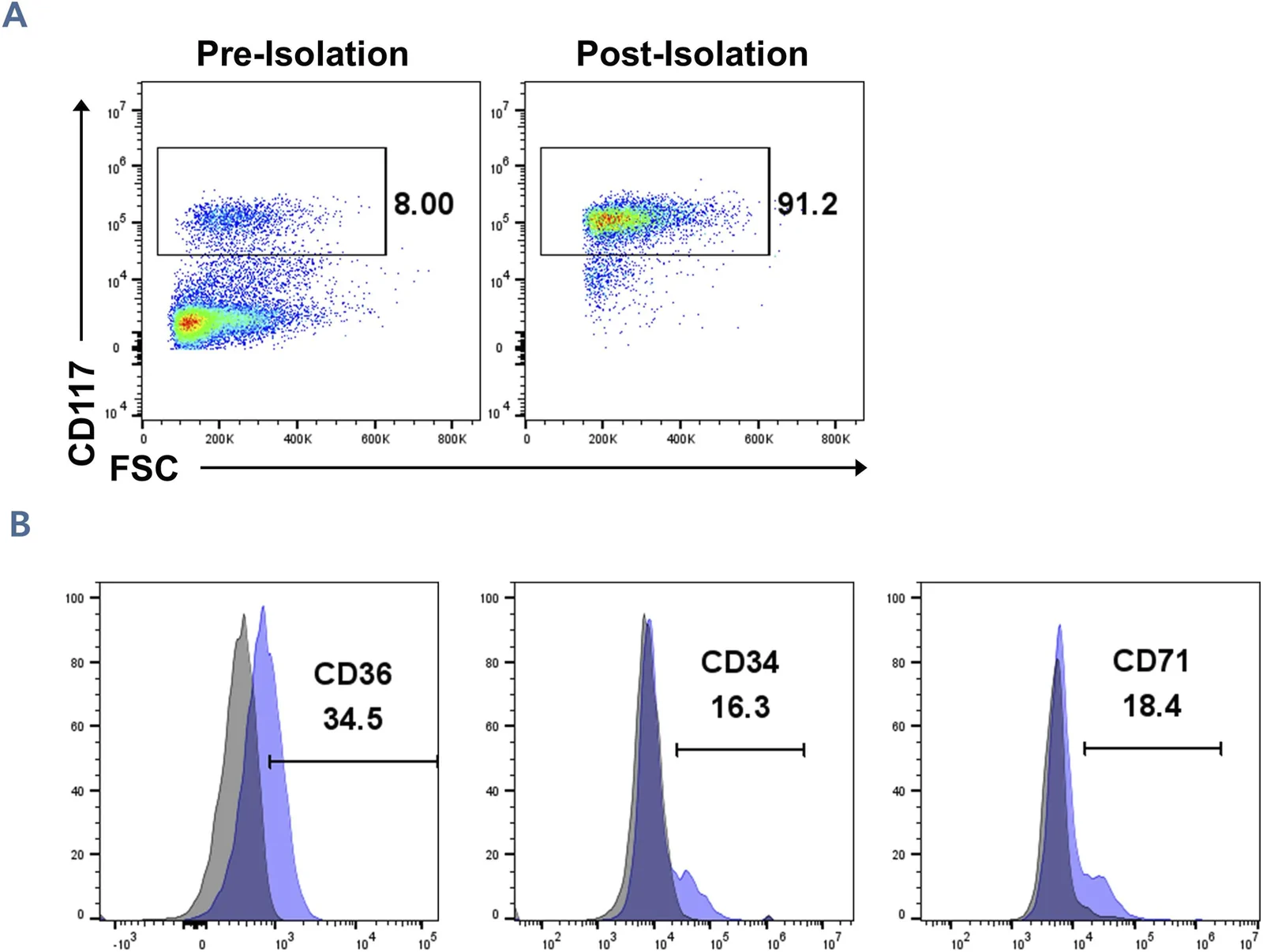
Enrichment and characterization of porcine CD117+ cells from Bone Marrow Mononuclear Cells. Hematopoietic progenitor cells (HPCs) expressing CD117 were isolated from pig bone marrow mononuclear cells (BM MNCs) using mouse anti-pig CD117 IgG antibody and anti-mouse IgG microbeads. **(A)** Flow cytometry was employed to quantify the percentage of CD117+ cells using PE-conjugated anti-mouse IgG antibody. **(B)** Isolated CD117+ cells were stained with anti-CD34, CD36 and CD71 antibodies and the expression levels were analyzed using flow cytometry with isotype control (gray).

Primary bone marrow mononuclear cells (BM MNCs) were isolated from the femur of healthy transgenic pigs (GGTA1/CMAH/B4galNT2/A3GalT2 Knockout and human CD46/TBM Knockin). Based on previous reports demonstrating effective enrichment of CD117^+^ hematopoietic stem/progenitor cells (HSPCs) that have the capacity to differentiate into erythroid lineages (Pérez et al., 2007), we first evaluated the proportion of CD117^+^ cells in porcine BM MNCs by flow cytometry (Figure 1A). CD117^+^ cells represented approximately 8% of the total BM MNC population. Following magnetic-activated cell sorting (MACS), the purity of the CD117^+^ fraction increased to over 90%, as confirmed by flow cytometry. To further characterize the sorted CD117^+^ population, we assessed the expression of erythroid-associated surface markers CD36, CD34, and CD71 (Figure 1B). CD36 is a membrane glycoprotein expressed on various cell types, including erythroid progenitors, and has been associated with colony-forming unit-erythroid (CFU-E) differentiation and expansion (De Wolf et al., 1994; Daniels et al., 2021). In the CD117^+^ population, 34.5% of cells expressed CD36, while CD34, a marker of hematopoietic progenitor cells, was expressed in 16.3% of the population. Additionally, CD71, the transferrin receptor involved in iron uptake during erythropoiesis, was expressed in approximately 18.4% of the CD117^+^ population. These findings indicate that the CD117^+^ population contains a heterogeneous mixture of early erythroid progenitor subsets, supporting its utility as a source for generating immortalized erythroid progenitor cell lines.

### 3.2 Generation of immortalized porcine erythroid progenitor cells from CD117+ bone marrow mononuclear cells

**FIGURE 2:**
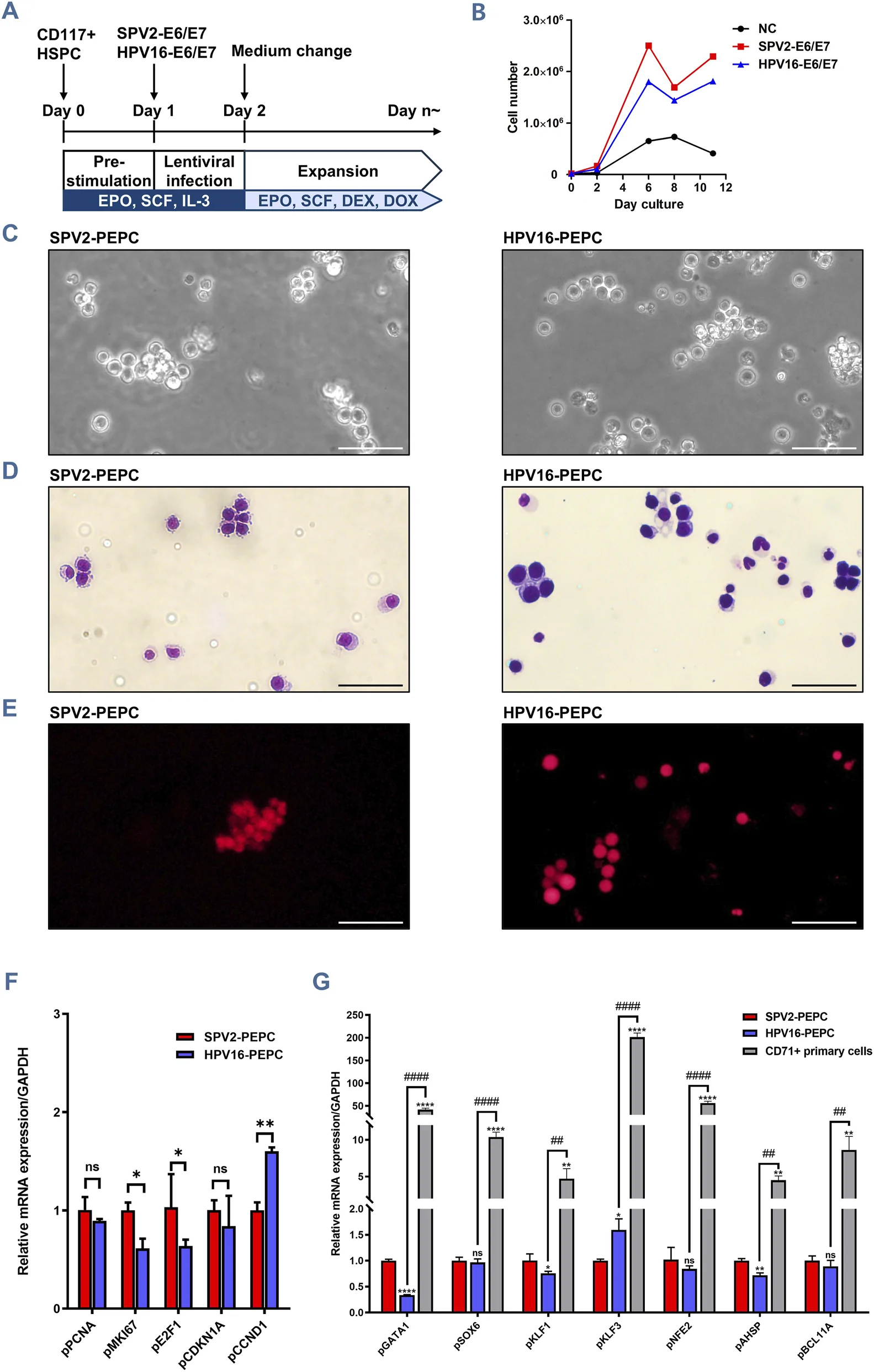
Morphological and proliferation analysis of immortalized porcine erythroid progenitor cell lines. **(A)** Schematic workflow illustrating the generation of immortalized porcine erythroid progenitor cell lines via lentiviral transduction with either SPV2 E6/E7 or HPV16 E6/E7 oncogenes. Abbreviations: HSPC, hematopoietic stem/progenitor cell; EPO, erythropoietin; SCF, stem cell factor; DEX, dexamethasone; DOX, doxycycline. **(B)** Growth curves comparing the proliferation of SPV2 E6/E7-transduced, HPV16 E6/E7-transduced, and non-transduced CD117^+^ cells over serial passages. Cell numbers were determined using trypan blue exclusion assays. **(C)** Representative bright-field images, **(D)** Wright-Giemsa staining and **(E)** fluorescence microscopy images of transduced cells showing hKO1 reporter expression of transduced cells, scale bars 50 µm. **(F)** Relative mRNA expression levels of key proliferation genes in immortalized cell line were quantified by qRT-PCR. P value was calculated using a two-tailed unpaired t-test (*p < 0.05, **p < 0.01, ****p < 0.001). **(G)** Relative mRNA expression levels of key erythroid transcription factors and lineage-specific genes in immortalized cell line were quantified by qRT-PCR. P value was calculated using a two-tailed unpaired t-test (*p < 0.05, **p < 0.01, ****p < 0.001 vs. SPV2-PEPC; ##p < 0.01, ####p < 0.001).

To establish an immortalized porcine erythroid progenitor cell line, we introduced E6 and E7 encoded by *Sus scrofa* papillomavirus (SPV2) and Human papillomavirus type 16 (HPV16) using Tet-inducible lentiviral vector system (Kurita et al., 2013; Daniels et al., 2021). The SPV2 E6/E7 coding sequence shares approximately 20%–30% similarity with HPV16 E6/E7 and retains key motifs, including CXXC, LXXLL or LXXLL-like, and LXCXE motifs (Online Supplementary Figure S1). As illustrated in Figure 2A, transduced cells were cultured in serum-free expansion medium. Proliferation analysis revealed that non-transduced cells followed the normal erythropoietic trajectory, undergoing terminal differentiation into enucleated red blood cells (RBCs), which was associated with a decline in proliferation capacity (Figure 2B). In contrast, cells transduced with either E6/E7 construct maintained their ability to proliferate (Figure 2B). Under these conditions, SPV2 E6/E7-transduced cells exhibited a significantly higher proliferation rate compared to those transduced with HPV16 E6/E7, suggesting enhanced proliferative capacity by the swine-derived oncogene pair under these culture conditions.

Cell viability and expansion were assessed using the trypan blue exclusion assay, confirming sustained growth in serum-free medium. Brightfield microscopy and Giemsa staining results showed that a large proportion of the transduced cells formed clusters resembling colony-forming unit-erythroid (CFU-E), consistent with early erythroid lineage characteristics (Figures 2C,D) (Hu et al., 2013). Additionally, the fluorescence microscopy images demonstrated strong expression of the hKO1 reporter, indicating successful transgene delivery and expression in both SPV2 and HPV16-transduced cells (Figure 2E).

We next examined the expression of several proliferation- and cell cycle-associated genes to further characterize the proliferative differences between SPV2-PEPC and HPV16-PEPC (Figure 2F). SPV2-PEPC exhibited significantly higher mRNA levels of MKI67 and E2F1, both of which are associated with cell cycle progression and mitotic activity (Mrouj et al., 2021; Yan et al., 2014). In contrast, no significant differences were observed in the expression of PCNA and CDKN1A, whereas CCND1 expression was reduced in SPV2-PEPC compared with HPV16-PEPC (Mrouj et al., 2021; Shen et al., 2025). Although these transcriptional changes do not fully explain the molecular mechanisms underlying SPV2-mediated immortalization, the elevated expression of MKI67 and E2F1 is partially consistent with the enhanced proliferative phenotype and colony-forming characteristics observed in Figure 2B, suggesting that SPV2-PEPC may possess a capacity for long-term expansion.

To assess the erythroid differentiation competence of the immortalized porcine erythroid progenitor line (PEPC) and compared with the primary CD71^+^ erythroid progenitor cells, we maintained the immortalized cells in continuous culture for more than 30 days and profiled key erythroid transcription factors by quantitative RT-PCR (Figure 2G). Quantitative analysis revealed that SPV2-PEPC, which are cells immortalized with SPV2 E6/E7, displayed markedly higher mRNA levels of GATA1, a master regulator of erythroid lineage commitment and terminal maturation, a KLF1-modulating transcription factor and AHSP, which stabilizes free α-globin during hemoglobin assembly (Fujiwara et al., 2004; Kihm et al., 2002; Dumitriu et al., 2010). In contrast, expression of β-globin-associated factor SOX6, the globin-switch regulator BCL11A, and NFE2, a transcription factor linked to hemoglobin biosynthesis and activation of the heme pathway, did not differ significantly between the two groups (Figure 2G) (Dumitriu et al., 2010; Trakarnsanga et al., 2014; Lecine et al., 1998). Notably, the expression of KLF3 was reduced in SPV2-PEPC compared with HPV16-PEPC (Figure 2G) (Funnell et al., 2007). The combination of elevated GATA1, KLF1 and AHSP together with decreased KLF3 suggests that SPV2 E6/E7 immortalization supports an erythroid transcriptional program that is favorable for erythroid maturation and hemoglobinization. Furthermore, the expression levels of these genes in primary CD71^+^ erythroid progenitor cells were substantially higher than those observed in both SPV2-PEPC and HPV16-PEPC, this difference may reflect distinct biological states of the primary and immortalized cells, as actively proliferating progenitors have been reported to transiently suppress erythroid differentiation programs while maintaining self-renewal capacity (Rosenbauer and Tenen, 2007; Ruijtenberg and van den Heuvel, 2016). However, when considered together with the erythroid transcription factor profiles shown in Figure 2G, these findings suggest that both immortalized cell lines retain proliferative capacity while preserving key erythroid progenitor-associated transcriptional programs. Therefore, the established cell lines appear to maintain a balance between self-renewal and erythroid lineage identity, supporting the potential utility of the immortalized porcine erythroid progenitor line (PEPC) for studies of porcine erythropoiesis and xenotransfusion.

### 3.3 Immunological characterization of immortalized porcine erythroid progenitor cells based on xenoantigen expression and human natural antibody binding

**FIGURE 3:**
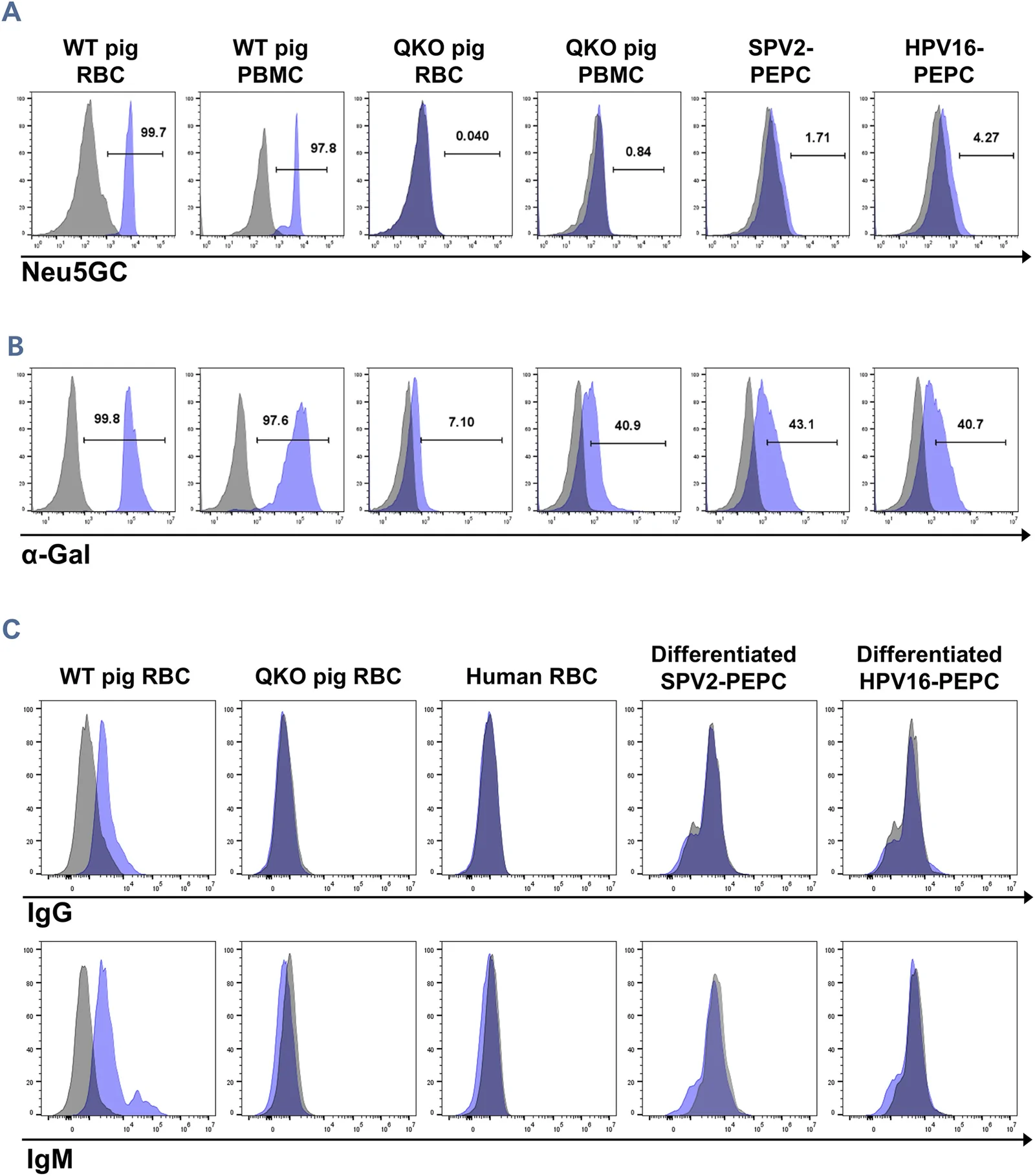
Immunological characterization of immortalized porcine erythroid progenitor cells based on xenoantigen expression and human natural antibody binding. **(A)** Representative flow cytometric histograms of Neu5Gc staining on red blood cells (RBC) and peripheral blood mononuclear cell (PBMC) from wild-type (WT) and quadruple knockout (QKO) pigs, alongside the immortalized porcine erythroid cell line. Cells were labeled with an anti-Neu5Gc antibody, and overlaid histograms for each population are shown as indicated with isotype control (gray). **(B)** Representative flow cytometric histograms of surface α-Gal staining using a biotin-conjugated GS-IB4 lectin specific for α-Gal. Overlaid histograms illustrate the distribution of α-Gal fluorescence intensity in WT RBC, WT PBMC, QKO RBC, QKO PBMC, and the immortalized erythroid cell lines with FITC secondary anti-biotin antibody control (gray). **(C)** Representative flow cytometric histograms showing human natural antibody binding to WT pig RBC, QKO pig RBC, human RBC, and differentiated immortalized porcine erythroid progenitor cell lines after incubation with 25% human serum. Surface-bound human IgG and IgM were detected using anti-human IgG and anti-human IgM antibodies.

Because α-Gal and Neu5Gc have been implicated as dominant xenoantigens on porcine cells in xenotransfusion models, we examined their surface expression on the newly generated erythroid cell line relative to RBC and PBMC from wild-type (WT) and QKO pigs. Neu5Gc was only detected in WT RBC and PBMC and was absent from QKO RBC, QKO PBMC, SPV2-PEPC, and HPV16-PEPC. As expected, α-Gal was highly expressed (>95% positive cells) in both WT RBC and WT PBMC (Figure 3A). In QKO RBC, α-Gal staining was restricted to a minor population of ∼7% of cells, whereas the immortalized cell line showed ∼40% α-Gal positivity, similar to QKO PBMC (Figure 3B). Collectively, these findings demonstrate that the xenoantigen expression pattern of the cell line mirrors that of QKO-based pig, with markedly diminished antigen levels relative to WT pig.

To further determine whether this reduced xenoantigen profile was associated with decreased recognition by human natural antibodies, WT pig RBCs, QKO pig RBCs, human RBCs, and differentiated PEPC-derived erythroid cells were incubated with 25% human serum, and surface-bound human IgG and IgM were analyzed by flow cytometry (Figure 3C). Representative histograms showed detectable human IgG and IgM binding only on WT pig RBCs. In contrast, QKO pig RBCs, human RBCs, and differentiated PEPC-derived erythroid cells showed little to no detectable binding of either human IgG or IgM. These results suggest that differentiated PEPC-derived erythroid cells exhibited low detectable human IgG and IgM binding under the tested *in vitro* conditions, similar to QKO pig RBCs.

### 3.4 Erythroid differentiation capacity of immortalized porcine erythroid progenitor cell line

**FIGURE 4:**
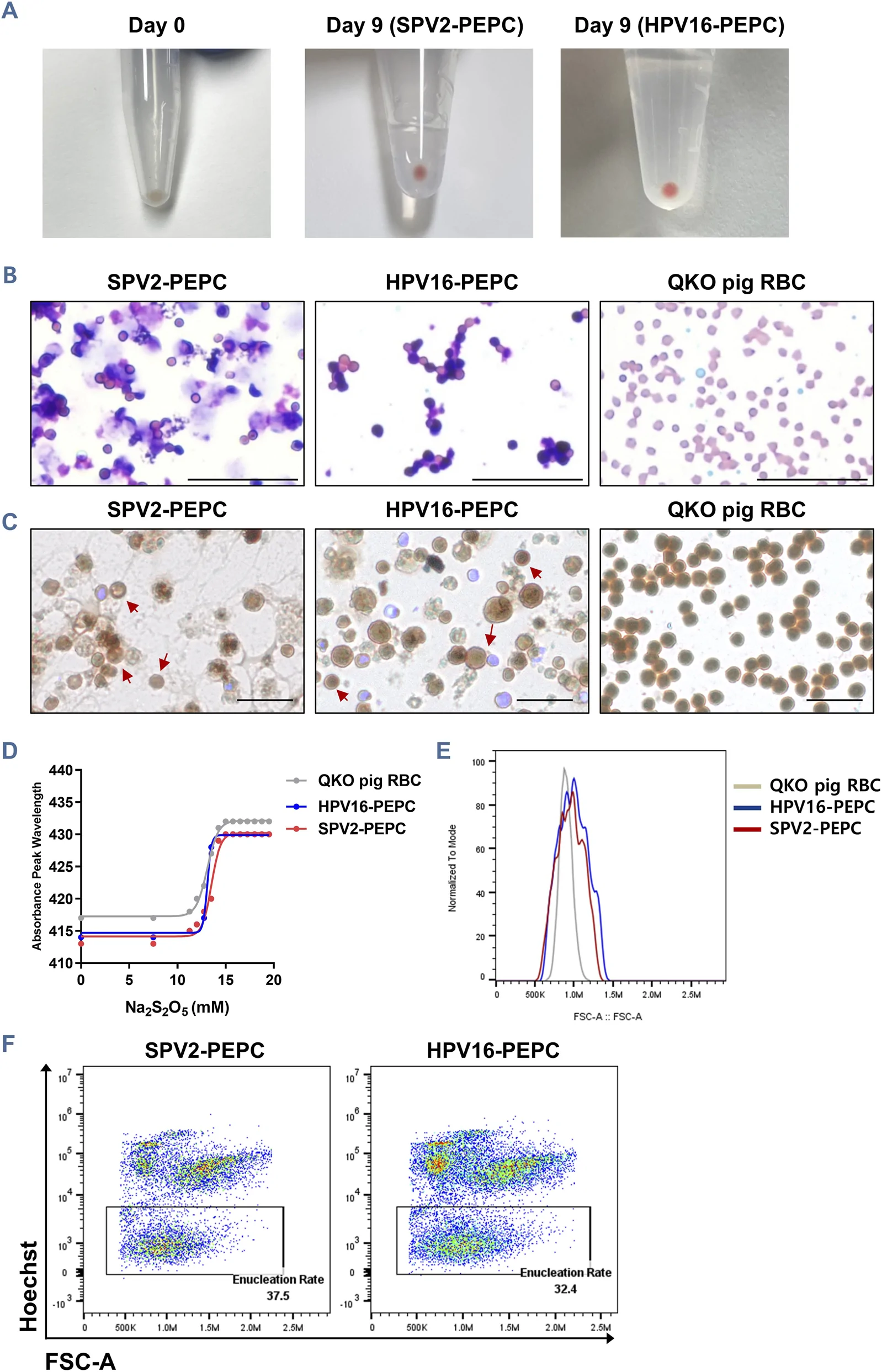
Morphological and functional analysis of differentiated immortalized porcine erythroid progenitor cell lines. **(A)** The induction of differentiation after 9 days is shown by red cell pellets, indicating hemoglobin synthesis in porcine erythroid cells. **(B)** Representative images showing morphology of differentiated cells after 18 days of culture in erythroid differentiation medium. Cells were stained with Wright-Giemsa and analyzed by light microscopy, scale bars 50 µm. **(C)** Benzidine staining of differentiated cells and QKO pig RBCs. Nucleated and enucleated cells were identified by Hoechst staining and analyzed by fluorescence microscopy. Red arrows indicate enucleated cells. Scale bars, 20 μm. **(D)** Hemoglobin-Oxygen curves of differentiated HPV16-PEPC and SPV2-PEPC cells compared with QKO pig RBCs derived from peripheral blood. **(E)** Flow cytometric analysis of forward scatter (FSC-A) demonstrating a reduction in cell size during erythroid differentiation. The differentiated cells exhibited a size distribution closer to that of mature porcine RBCs.​ **(F)** Representative flow cytometry plots showing the enucleation efficiency of differentiated SPV2-PEPC and HPV16-PEPC cells on day 14 following Hoechst staining.

To further evaluate their capacity for terminal erythroid maturation, PEPCs were placed in erythroid differentiation medium. After 9 days of differentiation, centrifugation of cultured cells revealed a distinct red pellet, indicating hemoglobin production and red blood cell generation, while undifferentiated controls produced a pale pellet (Figure 4A). Morphological assessment, together with benzidine staining to evaluate hemoglobin synthesis after 18 days of differentiation demonstrated the presence of enucleated cells that were morphologically comparable to mature porcine erythrocytes in peripheral blood (Figures 4B,C).

To assess oxygen transport capacity, hemoglobin-oxygen dissociation was measured in red blood cells fully differentiated from SPV2-PEPC and HPV16-PEPC and in QKO pig RBC (Figure 4D). Stepwise addition of sodium metabisulfite induced a progressive bathochromic shift of the Soret peak and a decrease in absorbance at ∼414 nm in both populations, consistent with gradual deoxygenation of hemoglobin (Sekyonda et al., 2024). The oxygen dissociation curves of differentiated RBCs from PEPC were sigmoidal and closely overlapped with that of QKO pig RBC, indicating preserved oxygen binding (Figure 4D). Flow cytometric analysis of forward scatter (FSC-A) demonstrated that the differentiated PEPC-derived cells exhibited a cell size distribution comparable to that of mature QKO pig RBCs (Figure 4E), indicating progressive maturation during erythroid differentiation. Furthermore, Hoechst staining followed by flow cytometric analysis revealed enucleation rates of 37.5% and 32.4% in SPV2-E6/E7 and HPV16-E6/E7 immortalized cells, respectively (Figure 4F). Together with the comparable hemoglobin-oxygen dissociation profiles, these data indicate that PEPC-derived erythrocytes acquire morphological and functional properties resembling those of peripheral pig RBCs.

**FIGURE 5:**
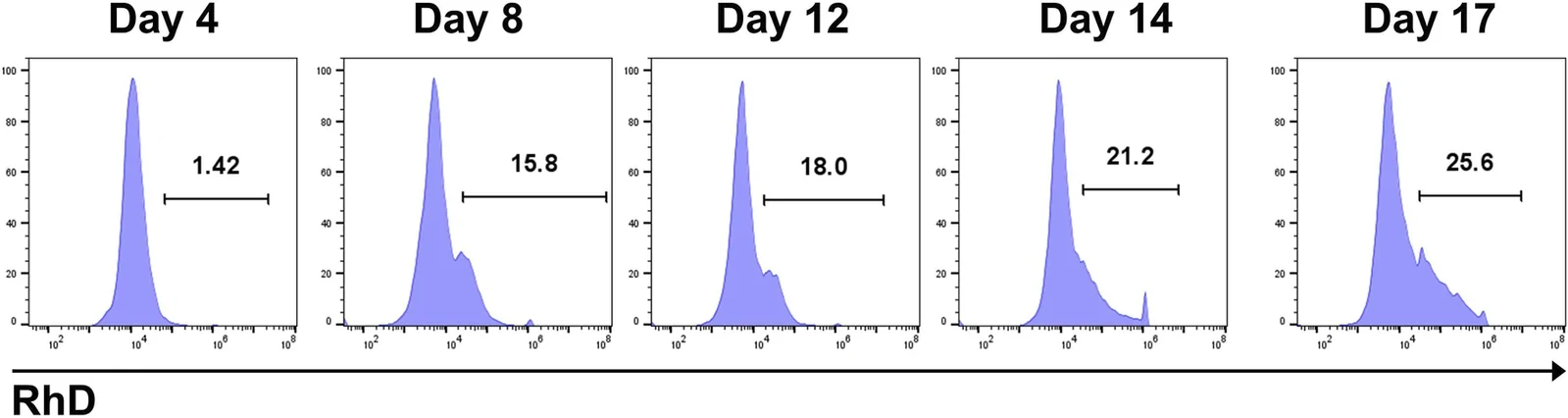
Flow cytometric analysis of cell surface protein expression during erythroid differentiation of SPV2-PEPC. SPV2-PEPC was cultured in erythroid differentiation medium following withdrawal of doxycycline. At days 4, 8, 12, 14, and 17, cells were harvested and stained with anti-RhD antibody, and analyzed by flow cytometry. Histograms show RhD surface expression at each time point, and the proportion of RhD-positive cells was quantified over the course of maturation.

During RBC induction of SPV2-PEPC, flow cytometry analysis revealed increased expression of the RhD antigen, a transmembrane protein marker of mature erythroid cells (Hu et al., 2013), confirming progression through erythroid maturation stages (Figure 5). These findings demonstrate the cell line’s ability to undergo terminal differentiation and synthesize hemoglobin, underscoring its utility as a model system for studying porcine erythropoiesis and red blood cell development.

## 4 Discussion

In this study, we report the establishment of the first immortalized porcine erythroid progenitor cell line derived from adult bone marrow CD117^+^ cells using papillomavirus E6/E7 oncogenes. This work addresses a critical gap in hematology and xenotransfusion research by providing a genetically tractable and renewable *in vitro* platform for studying porcine erythropoiesis, RBC maturation, and cross-species immune interactions (Butler et al., 2015; Smood et al., 2019; Park et al., 2023).

A key strength of this study lies in the choice of the starting cell population. Unlike previous erythroid cell line models that relied on CD34^+^ or CD36^+^ cells derived from cord blood, peripheral blood, or iPSC-based progenitors (Trakarnsanga et al., 2017; Kurita et al., 2013; Wong et al., 2010; Bagchi et al., 2021), we utilized CD117^+^ bone marrow-derived cells, which encompass a heterogeneous pool of early hematopoietic and erythroid progenitors (Pérez et al., 2007). Flow cytometric analysis confirmed co-expression of CD34, CD36, and CD71, indicating that this population spans multiple early erythroid differentiation stages (Leberbauer et al., 2005; De Wolf et al., 1994; Daniels et al., 2021). Preserving such heterogeneity is advantageous, as immortalization at earlier progenitor stages has been shown to retain proliferative plasticity while still allowing downstream erythroid maturation under appropriate differentiation conditions (Daniels et al., 2021).

The comparison between HPV16 E6/E7 and SPV2 E6/E7 represents an important conceptual advance. While HPV16 E6/E7-mediated immortalization is well established in human erythroid systems (Kurita et al., 2013), porcine cells appear to exhibit partial resistance to heterologous viral oncogenes, necessitating species-adapted strategies (Link et al., 2017). In this context, the long-term proliferation and transcriptional erythroid profile observed in SPV2 E6/E7-transduced cells highlight the importance of species-specific oncogenic drivers, the proliferative capacity of SPV2 E6/E7-transduced cells was accompanied by increased expression of MKI67 and E2F1, which are involved in regulating cell cycle progression and mitotic activity. Notably, SPV2-immortalized cells displayed higher expression of GATA1, KLF1 and AHSP, suggesting that SPV2 E6/E7 not only supports cell cycle progression but also preserves or enhances erythroid lineage identity (Fujiwara et al., 2004; Kihm et al., 2002; Dumitriu et al., 2010; Lecine et al., 1998). These findings underscore the broader relevance of species-adapted viral tools for large-animal cell line development.

The elevated expression of NFE2 in SPV2 E6/E7-immortalized cells is particularly noteworthy. NFE2 has been implicated in delaying premature erythroid maturation while promoting progenitor expansion, providing a plausible mechanistic explanation for the enhanced proliferative capacity observed in the SPV2 group (Lecine et al., 1998). At the same time, sustained expression of canonical erythroid transcription factors, including KLF1, KLF3, GATA1, BCL11A, and SOX6, supports the conclusion that immortalization did not abrogate erythroid commitment. The relatively lower expression of erythroid-associated genes in both immortalized cell lines compared with primary CD71^+^ cells may reflect their preferential commitment to proliferation, consistent with the well-established inverse relationship between cell proliferation and terminal erythroid differentiation reported in previous studies (Rosenbauer and Tenen, 2007; Ruijtenberg and van den Heuvel, 2016). Importantly, these genes remained detectable in both cell lines, indicating retention of erythroid progenitor characteristics following immortalization (Dumitriu et al., 2010; Funnell et al., 2014). Consistent with this, upon induction with erythroid differentiation medium, the established cell line demonstrated hallmarks of terminal erythroid maturation, including increased RhD expression, hemoglobin accumulation, progressive cell size reduction, and visible red pellet formation (Hu et al., 2013).

The human serum antibody-binding assay further supports the relevance of differentiated PEPC-derived erythroid cells for evaluating human natural antibody binding to genetically modified porcine erythroid cells. These findings suggest that PEPC-derived erythroid cells show a low human IgG and IgM antibody-binding profile similar to QKO pig RBCs under the tested *in vitro* conditions. From a translational perspective, this porcine erythroid progenitor cell line offers several advantages over transgenic pig models. The generation and maintenance of genetically modified pigs are costly and time-consuming, limiting their utility for high-throughput mechanistic studies (Butler et al., 2015; Smood et al., 2019). In contrast, the *in vitro* system described here enables scalable genetic manipulation, functional assays, and screening approaches relevant to xenotransfusion. In particular, this platform may facilitate systematic investigation of macrophage-mediated clearance mechanisms, including the balance of ‘eat me’ and ‘do not eat me’ signals such as CD47-SIRPα incompatibility, which has been shown to contribute to rapid xenogeneic cell clearance and macrophage-mediated rejection when porcine CD47 fails to engage human SIRPα (Ide et al., 2007; Wang et al., 2011), and is a major focus in xenotransplantation research (Navarro-Alvarez and Yang, 2011).

Several limitations of the current study should be acknowledged. First, due to limited availability of porcine-specific antibodies, comprehensive surface phenotyping of late-stage erythroid markers could not be performed. Second, functional properties of the generated red blood cells, including oxygen-binding capacity, detailed hemoglobin isoform composition, and deformability, were not directly assessed and will require future validation. Third, although SPV2 E6/E7 enabled efficient immortalization, the oncogenic mechanisms and long-term genetic stability of these cells require further investigation, particularly in the context of potential translational applications (Link et al., 2017). Fourth, the human serum antibody-binding assay only assessed surface-bound IgG and IgM *in vitro* and does not fully represent broader xenotransfusion-related immune barriers, such as complement activation, macrophage-mediated clearance, endothelial interactions, or *in vivo* survival in xenotransfusion model. Future studies incorporating karyotypic analysis, bulk RNA sequencing, and functional erythrocyte assays will be essential to fully define the utility and safety of this model.

In conclusion, we have established a novel immortalized porcine erythroid progenitor cell line using species-adapted papillomavirus oncogenes. This platform provides a valuable experimental system for studying porcine erythropoiesis, comparative hematology, and xenotransfusion-related immune barriers, and lays the groundwork for scalable *in vitro* production and genetic engineering of porcine RBCs for research and biotechnological applications.

## Data Availability

The original contributions presented in the study are included in the article/Supplementary Material. Further inquiries can be directed to the corresponding author.

## References

[B1] Bagchi A. Nath A. Thamodaran V. Ijee S. Palani D. Rajendiran V. (2021). Direct generation of immortalized erythroid progenitor cell lines from peripheral blood mononuclear cells. Cells 10, 1–18. 10.3390/CELLS10030523 33804564 PMC7999632

[B2] Butler J. R. Ladowski J. M. Martens G. R. Tector M. Tector A. J. (2015). Recent advances in genome editing and creation of genetically modified pigs. Int. J. Surg. 23, 217–222. 10.1016/j.ijsu.2015.07.684 26231992

[B3] Daniels D. E. Ferguson D. C. J. Griffiths R. E. Trakarnsanga K. Cogan N. MacInnes K. A. (2021). Reproducible immortalization of erythroblasts from multiple stem cell sources provides approach for sustainable RBC therapeutics. Mol. Ther. Methods Clin. Dev. 22, 26–39. 10.1016/j.omtm.2021.06.002 34485592 PMC8390520

[B4] De Wolf J. T. M. Muller E. W. Hendriks D. H. Halie R. M. Vellenga E. (1994). Mast cell growth factor modulates CD36 antigen expression on erythroid progenitors from human bone marrow and peripheral blood associated with ongoing differentiation. Blood 84, 59–64. 10.1182/BLOOD.V84.1.59.59 7517219

[B5] Douay L. (2018). Why industrial production of red blood cells from stem cells is essential for tomorrow’s blood transfusion. Regen. Med. 13, 13–632. 10.2217/RME-2018-0025 30246611

[B6] Dumitriu B. Bhattaram P. Dy P. Huang Y. Quayum N. Jensen J. (2010). Sox6 is necessary for efficient erythropoiesis in adult mice under physiological and anemia-induced stress conditions. PLoS One 5, e12088. 10.1371/JOURNAL.PONE.0012088 20711497 PMC2918505

[B7] Ellingson K. D. Sapiano M. R. P. Haass K. A. Savinkina A. A. Baker M. L. Chung K. W. (2017). Continued decline in blood collection and transfusion in the United States-2015. Transfusion 57 (Suppl. 2), 1588–1598. 10.1111/TRF.14165 28591469 PMC5556921

[B8] Fujiwara Y. Chang A. N. Williams A. M. Orkin S. H. (2004). Functional overlap of GATA-1 and GATA-2 in primitive hematopoietic development. Blood 103, 583–585. 10.1182/BLOOD-2003-08-2870 14504093

[B9] Funnell A. P. W. Maloney C. A. Thompson L. J. Keys J. Tallack M. Perkins A. C. (2007). Erythroid Krüppel-like factor directly activates the basic Krüppel-like factor gene in erythroid cells. Mol. Cell Biol. 27, 2777–2790. 10.1128/MCB.01658-06 17283065 PMC1899893

[B10] Funnell A. P. W. Vernimmen D. Lim W. F. Mak K. S. Wienert B. Martyn G. E. (2014). Differential regulation of the α-globin locus by Krüppel-like factor 3 in erythroid and non-erythroid cells. BMC Mol. Biol. 15, 8. 10.1186/1471-2199-15-8 24885809 PMC4033687

[B11] Hu J. Liu J. Xue F. Halverson G. Reid M. Guo A. (2013). Isolation and functional characterization of human erythroblasts at distinct stages: implications for understanding of normal and disordered erythropoiesis *in vivo* . Blood 121, 3246–3253. 10.1182/BLOOD-2013-01-476390 23422750 PMC3630836

[B12] Ide K. Wang H. Tahara H. Liu J. Wang X. Asahara T. (2007). Role for CD47-SIRPalpha signaling in xenograft rejection by macrophages. Proc. Natl. Acad. Sci. U. S. A. 104, 5062–5066. 10.1073/pnas.0609661104 17360380 PMC1829264

[B13] Kihm A. J. Kong Y. Hong W. Russell J. E. Rouda S. Adachl K. (2002). An abundant erythroid protein that stabilizes free alpha-haemoglobin. Nature 417, 758–763. 10.1038/NATURE00803 12066189

[B14] Kurita R. Suda N. Sudo K. Miharada K. Hiroyama T. Miyoshi H. (2013). Establishment of immortalized human erythroid progenitor cell lines able to produce enucleated red blood cells. PLoS One 8, 8. 10.1371/JOURNAL.PONE.0059890 23533656 PMC3606290

[B15] Leberbauer C. Boulmé F. Unfried G. Huber J. Beug H. Müllner E. W. (2005). Different steroids co-regulate long-term expansion versus terminal differentiation in primary human erythroid progenitors. Blood 105, 85–94. 10.1182/BLOOD-2004-03-1002 15358620

[B16] Lecine P. Blank V. Shivdasani R. (1998). Characterization of the hematopoietic transcription factor NF-E2 in primary murine megakaryocytes. J. Biol. Chem. 273, 7572–7578. 10.1074/jbc.273.13.7572 9516460

[B17] Link E. K. Hoferer M. Strobe B. Rigbers K. Langenmayer M. C. Sutter G. (2017). *Sus scrofa* papillomavirus 2 - genetic characterization of a novel suid papillomavirus from wild boar in Germany. J. Gen. Virol. 98, 2113–2117. 10.1099/JGV.0.000868 28758619

[B18] Mrouj K. Andrés-Sánchez N. Dubra G. Singh P. Sobecki M. Chahar D. (2021). Ki-67 regulates global gene expression and promotes sequential stages of carcinogenesis. Proc. Natl. Acad. Sci. U. S. A. 118 (10), e2026507118. 10.1073/pnas.2026507118 33658388 PMC7958263

[B19] Navarro-Alvarez N. Yang Y. G. (2011). CD47: a new player in phagocytosis and xenograft rejection. Cell Mol. Immunol. 8, 285–288. 10.1038/cmi.2010.83 21258362 PMC3644051

[B20] Ohshima S. Mori S. Shigenari A. Miyamoto A. Takasu M. Imaeda N. (2014). Differentiation ability of multipotent hematopoietic stem/progenitor cells detected by a porcine specific anti-CD117 monoclonal antibody. Biosci. Trends 8, 308–315. 10.5582/BST.2014.01084 25641176

[B21] Park S. Lee H. Park E. M. Roh J. Kang P. I. Shim J. (2023). Initial investigation on the feasibility of porcine red blood cells from genetically modified pigs as an alternative to human red blood cells for transfusion. Front. Immunol. 14, 14. 10.3389/FIMMU.2023.1298035 38035112 PMC10682702

[B22] Pérez C. Moreno S. Summerfield A. Domenech N. Alvarez B. Correa C. (2007). Characterisation of porcine bone marrow progenitor cells identified by the anti-c-kit (CD117) monoclonal antibody 2B8/BM. J. Immunol. Methods 321, 70–79. 10.1016/j.jim.2007.01.003 17313957

[B23] Rosenbauer F. Tenen D. G. (2007). Transcription factors in myeloid development: balancing differentiation with transformation. Nat. Rev. Immunol. 7, 105–117. 10.1038/nri2024 17259967

[B24] Ruijtenberg S. van den Heuvel S. (2016). Coordinating cell proliferation and differentiation: antagonism between cell cycle regulators and cell type-specific gene expression. Cell Cycle 15, 196–212. 10.1080/15384101.2015.1120925 26825227 PMC4825819

[B25] Scully E. J. Shabani E. Rangel G. W. Grüring C. Kanjee U. Clark M. A. (2019). Generation of an immortalized erythroid progenitor cell line from peripheral blood: a model system for the functional analysis of plasmodium spp. invasion. Am. J. Hematol. 94, 963–974. 10.1002/AJH.25543 31148215 PMC6984401

[B26] Sekyonda Z. An R. Goreke U. Man Y. Monchamp K. Bode A. (2024). Rapid measurement of hemoglobin-oxygen dissociation by leveraging bohr effect and soret band bathochromic shift. Analyst 149, 2561–2572. 10.1039/D3AN02071A 38501195 PMC11056771

[B27] Shen W. Duan F. Fu S. Cui P. Shi Y. Zhang L. (2025). CDKN1A and cellular senescence are associated with immune resistance in advanced lung adenocarcinoma. Clin. Exp. Med. 26 (1), 14. 10.1007/s10238-025-01926-2 41249682 PMC12628481

[B28] Smood B. Hara H. Schoel L. J. Cooper D. K. C. (2019). Genetically-engineered pigs as sources for clinical red blood cell transfusion: what pathobiological barriers need to be overcome? Blood Rev. 35, 7–17. 10.1016/j.blre.2019.01.003 30711308 PMC6467751

[B29] Trakarnsanga K. Wilson M. C. Lau W. Singleton B. K. Parsons S. F. Sakuntanaga P. (2014). Induction of adult levels of β-globin in human erythroid cells that intrinsically express embryonic or fetal globin by transduction with KLF1 and BCL11A-XL. Haematologica 99, 1677–1685. 10.3324/HAEMATOL.2014.110155 25107887 PMC4222483

[B30] Trakarnsanga K. Griffiths R. E. Wilson M. C. Blair A. Satchwell T. J. Meinders M. (2017). An immortalized adult human erythroid line facilitates sustainable and scalable generation of functional red cells. Nat. Commun. 8, 8. 10.1038/NCOMMS14750 28290447 PMC5355882

[B31] Wang C. Wang H. Ide K. Wang Y. van Rooijen N. Ohdan H. (2011). Human CD47 expression permits survival of porcine cells in immunodeficient mice that express SIRPα capable of binding to human CD47. Cell Transpl. 20, 1915–1920. 10.3727/096368911X566253 21535911 PMC3670959

[B32] Wang Z. Y. Burlak C. Estrada J. L. Li P. Tector M. F. Tector A. J. (2014). Erythrocytes from GGTA1/CMAH knockout pigs: implications for xenotransfusion and testing in non-human primates. Xenotransplantation 21, 376–384. 10.1111/XEN.12106 24986655 PMC4366650

[B33] Wong S. Keyvanfar K. Wan Z. Kajigaya S. Young N. S. Zhi N. (2010). Establishment of an erythroid cell line from primary CD36+ erythroid progenitor cells. Exp. Hematol. 38, 994–1005. 10.1016/j.exphem.2010.07.012 20696208 PMC6341492

[B34] Yan L. H. Wei W. Y. Cao W. L. Zhang X. S. Xie Y. B. Xiao Q. (2014). Overexpression of E2F1 in human gastric carcinoma is involved in anti-cancer drug resistance. BMC Cancer 14 (1), 904. 10.1186/1471-2407-14-904 25466554 PMC4258940

[B35] Zhu A. (2000). Introduction to porcine red blood cells: implications for xenotransfusion. Semin. Hematol. 37, 143–149. 10.1016/S0037-1963(00)90039-8 10791883

