# Supplementary material for: Establishment of an immortalized porcine erythroid progenitor cell line using species-adapted papillomavirus oncogenes
Source: Front Bioeng Biotechnol. 2026 Aug 26;14:1807068. doi: 10.3389/fbioe.2026.1807068 (PMC13553742; doi:10.3389/fbioe.2026.1807068)
Supplement: Supplementary file 1 [file Supplementaryfile1.docx]

Supplementary Material

**Supplementary Figure 1.** **Comparative alignment of E6 and E7 amino acid sequences from human papillomavirus type 16 and porcine papillomavirus type 2.** Amino acid sequences of E6 and E7 proteins from HPV16 and SPV2. The accession numbers for the sequences are E6, E7 (KY817993.1) for pig and E6 (NP_041325), E7 (NP_041326) for human. The stars below the sequences indicate conserved amino acids, CXXC motifs are highlighted in green, and LXXLL, LXXLL-like and LXCXE motifs are highlighted in blue.

| **Medium** | **Supplements** |
| --- | --- |
| Stimulation | IMDM (Gibco)  + 3% heat-inactivated porcine serum (Gibco)  + 2% heat-inactivated fetal bovine serum (Gibco)  + 3U/ml heparin (Sigma-Aldrich)  + 10ug/ml porcine insulin (Cell Applications)  + 3Uml porcine EPO (RayBiotech Life)  + 10ng/ml human SCF (STEMCELL technologies)  + 1ng/ml porcine IL-3 (R&D systems) |
| Expansion | StemSpan SFEM II (STEMCELL technologies)  + 3U/ml porcine EPO (RayBiotech Life)  + 50ng/ml human SCF (STEMCELL technologies)  + 1uM DEX (Sigma-Aldrich)  + 1ug/ml DOX (Takara) |
| Differentation | IMDM (Gibco)  + 3% heat-inactivated porcine serum (Gibco)  + 2% heat-inactivated fetal bovine serum (Gibco)  + 3U/ml heparin (Sigma-Aldrich)  + 10ug/ml porcine insulin (Cell Applications)  + 3Uml porcine EPO (RayBiotech Life)  + 500ug/ml human holo-transferrin (Sigma-Aldrich) |

**Supplementary Table S1. Medium composition**

Abbreviations: EPO, erythropoietin; SCF, stem cell factor; DEX, dexamethasone; DOX, doxycycline.

| **Porcine Gene** | **Primer name** | **Sequence (5' → 3')** |
| --- | --- | --- |
| GATA1 | pGATA1_Forward | GGTACAGGAGCAGCTGAAGG |
|  | pGATA1_Reverse | TGGTAGAGGTGGGCAGTACC |
| SOX6 | pSOX6_Forward | TCCCCATCTCCTAACCACAG |
|  | pSOX6_Reverse | GAACTGTCCCTTTCGCTGAC |
| KLF1 | pKLF1_Forward | CCACATGTCCTTGACCACAG |
|  | pKLF1_Reverse | AGGGTCTCTGACAAGCCTCA |
| KLF3 | pKLF3_Forward | GTTGAGGGGATGGATGCT |
|  | pKLF3_Reverse | AGACAGGTGGGTGGATGAC |
| NFE2 | pNFE2_Forward | CAGAGCAGGAACAGGGTGA |
|  | pNFE2_Reverse | GGATTGAGCAGGGGCAGTA |
| AHSP | pAHSP_Forward | CAGGAAGAGCAGGACAAGG |
|  | pAHSP_Reverse | ATAACACCCCGAAAAAGCAG |
| BCL11A | pBCL11A_Forward | CTCTCCTCCCCTCGTTCTG |
|  | pBCL11A_Reverse | GACTTCCGTGTTCGCTTTCTA |
| GAPDH | pGAPDH_Forward | ACCCAGAAGACTGTGGAT |
|  | pGAPDH_Reverse | GTCCTCAGTGTAGCCCAGGA |
| MKI67 | p MKI67_Forward | CTGCGGATGGAGGAGAGATT |
|  | p MKI67_Reverse | AGGACCAGTGAATGAAGGCT |
| PCNA | pPCNA_Forward | AGCCACTCCACTCTCTCCTA |
|  | pPCNA_Reverse | GCATCACCGAAGCAGTTCTC |
| CCND1 | p CCND1_Forward | CTGGGTGATTGGAATGGCTT |
|  | p CCND1_Reverse | CTTGGCGGAGATTTGGAGTT |
| E2F1 | pE2F1_Forward | CCTCCTGTGGCATTTGAAGT |
|  | pE2F1_Reverse | AACCAAAGCAGAGGGAACAG |
| CDKN1A | pCDKN1A_Forward | CTGTCCAGTGAAAGTAGGGC |
|  | pCDKN1A_Reverse | ATGGAGAAGGGTAGAGGAGC |

**Supplementary Table S2.** Primers used for qRT-PCR

**Table S3**. **List of Materials to detect cell surface molecules used in this study**

| Target | Detector | Supplier | Catalog |
| --- | --- | --- | --- |
| CD34 | Antibody (clone EP373Y) | Abcam | ab198395 |
| CD117 | Antibody (clone 2B8/BM) | MyBioSource | MBS224470 |
| CD36 | Antibody (clone CB38) | BD Biosciences | 555454 |
| CD71 | Antibody (epitope aa578-765) | LSBio | LS-C417956 |
| Neu5Gc | Antibody (clone Poly21469) | Biolegend | 146901 |
| α-Gal epitope | Isolectin B_4_ (BSI-B_4_) | Sigma-Aldrich | L2140-.2MG |
| RhD | Antibody (clone C2) | Cloud-Clone | MAP729Hu21 |
| Human IgG | Antibody (Clone M1310G05) | Biolegend | 410710 |
| Human IgM | Antibody (Clone MHM-88) | Biolegend | 314511 |
